# Croup and Diptheria

**Published:** 1875-11

**Authors:** 


Croup and Diphtheria.—The eminent surgeon, M. James Spence, has the following remarks in a recent address:

In speaking of operations in croup, I have used the terms simple and diphtheritic croup; and I have done so advisedly, because, whilst the average results of my operations have been as good in the one disease as in the other, I consider them as essentially different diseases, and I do not believe that an extended experience would give the same amount of success in diphtheritic as in simple croup. It has been with no small amazement I have read some of the views recently propagated, that croup and diphtheritic croup are identical. I can hardly conceive two diseases more different, whether we consider them in their causation, symptoms, or sequlæ. In one feature, doubtless, there is similarity, because when, in diphtheria, the air-passages become affected, the presence of the membrane exuded necessarily gives rise to the same physical symptoms as to sound of voice, breathing, and asphyxiating paroxysms, as the false membrane in simple croup does. But in diphtheria the exudations in the larynx or elsewhere are local expression of a special blood-disease, which may, and often does, destroy life without affecting the air-passages at all, whereas, in simple croup, the false membrane is the result of a local inflammation. The cause or circumstances in which the two diseases originate are, according to my experience, very different. Ordinary croup almost invariably arises from exposure to cold, or occasionally from some source of local irritation, leading directly to inflammation of the mouth, as dentition. It is most frequent during cold moist weather, and specially during the prevalence of easterly or northeasterly winds. The late Professor Alison used to say that, according to his observation amongst the poorer classes, the affection most frequently occurred between Saturday night and Monday morning; and he attributed this to the custom of washing the floors of the rooms on the Saturday night, after the children were in bed. Diphtheria, on the other hand, prevails at all seasons and during all kinds of weather, sometimes as an epidemic, and then generally coincident with scarlet fever, but always more or less connected with, or influenced by, the effects of sewage emanations or imperfect drainage. Hence we meet with it more frequently amongst the better classes, and in houses with modern accommodations, such as fixed wash-basins and water closet accommodations in immediate connection with nurseries or bedrooms.

Diphtheria is undoubtedly infectious, both by direct contact of the sputa with a healthy mucous surface, as has been too often proved by members of our profession and by mothers, or by emanations from the affected person, as evidenced by the manner in which it spreads in a family. Simple croup, as I have been accustomed to see it, has no such contagious or infectious character. In dispensary practice, I have frequently seen a child affected with croup lying in a confined room amongst other children; but I never knew the disease to spread as diphtheria does. The peculiar nervous affection, the paralysis, which follows diphtheria, has counterpart in ordinary croup; nor, in cases of simple croup, were we accustomed see the white leatherly pedicle on the tonsils or fauces, though it was a very common disease in Edinburgh and its vicinity. I know that in France the fauces were always examined, and that false membranes or pedicles were considered symptomatic of croup; but that only leads me to believe that the disease in France was always a different type—diphtheritic, in fact.—*The Medical and Surgical Reporter.*

